# Shedding Light on the Effect of Natural Anti-Herpesvirus Alkaloids on SARS-CoV-2: A Treatment Option for COVID-19

**DOI:** 10.3390/v12040476

**Published:** 2020-04-23

**Authors:** Sherif T. S. Hassan

**Affiliations:** Department of Applied Ecology, Faculty of Environmental Sciences, Czech University of Life Sciences Prague, Kamýcká 129, 6-Suchdol, 16521 Prague, Czech Republic; sherif.hassan@seznam.cz; Tel.: +420-774-630-604

**Keywords:** herpesviruses, COVID-19, alkaloids, anti-herpesvirus drugs, SARS-CoV-2

## Abstract

The whole world is currently facing an unseen enemy, called coronavirus disease 2019 (COVID-19), which is causing a global pandemic. This disease is caused by a novel single-stranded enveloped RNA virus, known as the Severe Acute Respiratory Syndrome Coronavirus-2 (SARS-CoV-2). Although huge efforts are being made to produce effective therapies to combat this disease, it continues to be one of the greatest challenges in medicine. There is no doubt that herpesviruses are one of the most important viruses that infect humans and animals, and infections induced by these pathogens have developed into a great threat to public health. According to the currently available evidence, the correlation between herpesviruses and coronaviruses is limited to the induced complications following the infections. For instance, the inflammation that is induced at the sites of infection could tie these viruses to each other in a relationship. Another example, bovine herpesvirus 1, which is an important pathogen of cattle, can cause a severe respiratory infection; the same way in which SARS-CoV-2 affects humans. Considering the current circumstances related to the COVID-19 crisis, this editorial paper, which belongs to the Special Issue “Recent Advances in Herpesviruses Research: What’s in the Pipeline?” aims to draw attention to some natural anti-herpesvirus alkaloid compounds, which have recently been proven to have excellent inhibitory efficacy against SARS-CoV-2 replication. Thus, this special focus is an attempt to hunt down various treatment options to combat COVID-19 based on repurposing drugs that are known to have multiple antiviral properties, including against herpesvirus.

As of 15 April 2020, there are 1,914,916 confirmed infected cases present in 213 countries, areas, or territories around the world, along with 123,010 confirmed deaths, according to the World Health Organization [1]. This outbreak shows us the urgent need to control this disease. Considering that several attempts are ongoing to design proper vaccines and monoclonal antibodies against SARS-CoV-2, some other investigational treatments based on natural products that affect SARS-CoV-2 cell invasion and replication might be studied [2,3]. It is known that herpesvirus (double-stranded enveloped DNA viruses) diseases are contagious and have become a global concern due to their immense threat to public health [4]. These pathogens induce latent and lytic infections in humans and animals [5]. As observed with all viruses, herpesviruses and SARS-CoV-2 share the same characteristic in which host receptor recognition is an essential step for viral infection. Therefore, targeting these receptors could be a valuable approach for designing effective antiviral drugs [6,7].

Natural compounds have played, for decades, a central role in microbiology research, as they were involved in the treatment of various infectious diseases and/or served as templates for designing novel antimicrobial medications with diverse mechanisms of action [8,9]. In a recent study that has been published in the journal *Antiviral Research*, two natural alkaloid type compounds with notable anti-herpesvirus properties (homoharringtonine and emetine; Figure 1) were detected for their ability to effectively inhibit the replication of SARS-CoV-2 in vitro with EC_50_ (50% effective concentration) values of 2.55 and 0.46 µM, respectively [10]. However, the study did not disclose the mechanisms by which both compounds induced anti-SARS-CoV-2 activity. On the other hand, such outcomes might open the door to further investigations to optimize and design these compounds as effective drugs to combat COVID-19. 

Since we are all interested in gathering the latest scientific findings and knowledge regarding COVID-19 and its possible treatment, I would like to share with the readers of the journal *Viruses* some in-depth details about the above-mentioned compounds.

Homoharringtonine (recently known as omacetaxine mepesuccinate) is a natural alkaloid derived from some species of the *Cephalotaxus* genus and belongs to the class of cephalotaxus alkaloids [11]. This drug is a protein synthesis inhibitor and was approved by the U.S. Food and Drug Administration (FDA) for curing chronic or accelerated phase chronic myeloid leukemia [12]. In previous preclinical investigations, this compound was reported with powerful antiviral activity against herpesviruses such as herpes simplex virus 1, pseudorabies virus, and varicella-zoster virus [13,14].

Emetine is a natural alkaloid isolated from *Psychotria ipecacuanha* and belongs to the class of emetine alkaloids [15]. Emetine is a protein synthesis inhibitor used to treat amoebiasis. However, some unfavorable outcomes were reported to be associated with its use, including cardiotoxicity [16]. Its noticeable anti-herpes properties were described against herpes simplex virus 2, cytomegalovirus, and bovine herpesvirus 1 [17,18,19].

Notably, the comprehensive review article that has been published in the journal *Viruses* (Special Issue “Recent Advances in Herpesviruses Research: What’s in the Pipeline?”) by Treml et al. [20] documented a large number of natural bioactive molecules and macromolecules with anti-herpes simplex virus properties along with their mechanisms of action. Although this paper has focused on the bioactivity against herpes simplex viruses, we cannot rule out that the reviewed natural products might have additional antiviral effects on various types of coronavirus, including SARS-CoV-2. On the other hand, this valuable review article might serve as a template to hunt down potential compounds that may be considered in further preclinical studies for evaluation against SARS-CoV-2. To date, there are several promising treatments for COVID-19 under investigation, but none with proven clinical efficacy. Therefore, all efforts should be gathered to overcome this serious problem and save the lives of many people all over the world. 

Finally, special thanks should be given to all of the authors, reviewers, and editorial staff, who contributed to the Special Issue “Recent Advances in Herpesviruses Research: What’s in the Pipeline?” and made this issue conceivable, both at *Viruses* and in the broader academic community.

## Figures and Tables

**Figure 1:**
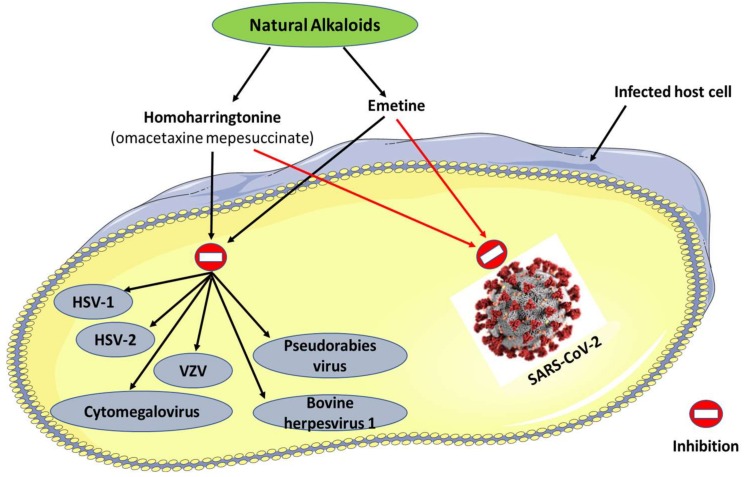
Natural alkaloids with dual anti-infective properties against SARS-CoV-2 and various types of herpesvirus. HSV: herpes simplex virus; VZV: varicella-zoster virus; SARS-CoV-2: Severe Acute Respiratory Syndrome Coronavirus-2.
